# Supporting addiction affected families effectively: a feasibility randomised controlled trial of a psychosocial intervention delivered by lay counsellors in Goa, India

**DOI:** 10.1017/gmh.2022.41

**Published:** 2022-08-26

**Authors:** Urvita Bhatia, Richard Velleman, Gill Velleman, Alison Garber, Alexander Catalano, Abhijit Nadkarni

**Affiliations:** 1 Sangath, Goa 403501, India; 2 Oxford Brookes University, Oxford, UK; 3 University of Bath, Bath, UK; 4 Brigham and Women's Hospital and the Harvard Graduate School of Education, 75 Francis St, Boston, MA 02115, USA; 5 London School of Hygiene and Tropical Medicine, London, UK

**Keywords:** Addiction, families, feasibility randomised controlled trial, India, intervention development, psychosocial intervention

## Abstract

**Background:**

Despite evidence of the burden of alcohol use on families, there is a lack of adequate and targeted support. We aimed to examine the feasibility, acceptability and impact of Supporting Addiction Affected Families Effectively (SAFE), a brief lay counsellor-delivered intervention for affected family members (AFMs).

**Methods:**

Parallel arm feasibility randomised controlled trial [1:1 allocation to SAFE or enhanced usual care (EUC)]. The primary outcome was mean difference in symptom score assessed by the Symptom Rating Test and secondary outcomes were difference in coping, impact and social support scores measured by the Coping Questionnaire, Family Member Impact Questionnaire, and Alcohol, Drugs and the Family Social Support Scale. Process data examining feasibility and acceptability were also collected. The primary analysis was intention to treat at the 3-month endpoint.

**Results:**

In total, 115 AFMs were referred to the trial, and 101 (87.8%) consenting participants were randomised to the two arms (51 SAFE arm and 50 EUC arm). Seventy-eight per cent completed treatment, with the mean number of sessions being 4.25 sessions and mean duration being 53 min. Ninety-five per cent completed outcome assessment. There were no statistically significant differences between SAFE and EUC on any of the outcome measures, except for the between-group adjusted mean differences for social support scores (AMD −6.05, 95% CI −10.98 to −1.12, *p* = 0.02).

**Conclusion:**

Our work indicates that it is possible to identify AFMs through community networking, and have high rates of participation for lay counsellor-delivered psychosocial care. Nevertheless, there is a need for further intervention development to ensure its contextual relevance and appropriateness.

## Introduction

Over 100 million family members worldwide are estimated to be affected by the addictive behaviours of a relative, and the negative impact is similar in family members across the world (Copello *et al*., 2010a, 2010b; Orford *et al*., 2013b). There has been a substantial increase in alcohol users over the years in India, with the annual alcohol consumption rates having increased by 38% between 2010 and 2017 (from 4.3 to 5.9 litres per adult) (Manthey *et al*., 2019). This increase will have caused a corresponding rise in the prevalence of affected family members (AFMs), and this will be largely hidden because AFMs are a ‘silent group’. The experiences of living with a person with drinking problems may make family members vulnerable to mental ill-health including mood and substance use disorders, trauma and stress-related conditions (Patel, 2007; Ray *et al*., 2009; Copello *et al*., 2010a, 2010b; Orford *et al*., 2013b). The limited number of studies from India demonstrate a high burden of alcohol use on families, including stress, disruptions in family interactions and routine, financial difficulties (Mattoo *et al*., 2013) and domestic violence and stigma (Patel *et al*., 2006; Gururaj *et al*., 2011).

Traditionally, family members have been viewed as a cause for the addiction, with the female spouse most often blamed (Orford *et al*., 2013a); and the focus of treatments for substance use is limited by engaging solely with the person with drinking-related problems (Copello *et al*., 2005). In India too, the treatment response has primarily focussed on long-term tertiary care for the person with drinking-related problems; which is fraught with further challenges of inaccessibility for the majority of the population (Benegal *et al*., 2009). Consequentially, AFMs experience high unmet needs as they play a peripheral role in formal care and are neglected because services are not equipped to address their problems (Copello and Orford, 2002; Orford *et al*., 2013b; Orford, 2017).

The 5-Step Method is an evidence-based brief psychosocial intervention for helping AFMs and is derived from the theoretical framework of the Stress-Strain-Coping-Support (SSCS) model (Velleman *et al*., 2008; Copello *et al*., 2009, 2010a, 2010b; Orford *et al*., 2010a, 2010b; Rey *et al*., 2010; Velleman *et al*., 2011; Orford *et al*., 2013a). The 5-Step Method was adapted and evaluated in the SAFE (Supporting Addiction Affected Families Effectively) program, using the Medical Research Council's guidance on developing and evaluating complex interventions (Craig *et al*., 2008; Rane *et al*., 2017; Church *et al*., 2018; Nadkarni *et al*., 2019). This paper describes the second phase of the SAFE program, and its objectives are to test the feasibility and acceptability of processes for a definitive randomised controlled trial (RCT) (e.g. recruitment, data collection, intervention delivery); test the indicative impact of the intervention in improving clinical outcomes in AFMs; and understand how the intervention can be further refined, based on these findings.

## Methods

### Study design

Parallel arm, single-blind, feasibility RCT. The CONSORT statement can be accessed in supplementary material.

### Setting

Communities in four sub-districts of Goa, India (population 1.4 million). Unlike most of India, Goa has a ‘more liberal, wet culture’ with the drinking patterns characterised by low rates of abstinence, and a high prevalence of hazardous drinking in men (Silva *et al*., 2003; D'costa *et al*., 2006; Pillai *et al*., 2013).

### Participants

The sources of recruitment were referrals from community gatekeepers (e.g. professional and community health workers, priests, village council members), and self-referrals (through word-of-mouth publicity and advertisements in healthcare and community settings). The inclusion criteria were (1) family member (⩾18 years) reporting that the relative's drinking had been a major source of distress in the previous 6 months, (2) the relative had been drinking problematically at some point during the previous 6 months, (3) the family member and the relative who had been drinking problematically had been living in the same house at some point in the previous 6 months, or had face-to-face contact at least three times a week. Eligible participants who gave informed consent were administered the baseline assessments and randomised to receive either SAFE or enhanced usual care (EUC).

### Randomisation, allocation concealment and blinding

An independent researcher developed a randomisation code using computer-generated random numbers. Sequentially Numbered Opaque Sealed Envelopes were used to maximise allocation concealment. The envelope was opened only after the participant information was written on its cover, which helped create an audit trail. The 3-month outcome assessments were administered by research assistants, who had no previous engagement in the trial, and were ‘blind’ to the treatment allocation. The primary outcome assessment was administered prior to all other assessments; and participants were requested not to disclose their allocation status during the outcome interview.

### Interventions

**Fig. 1.**
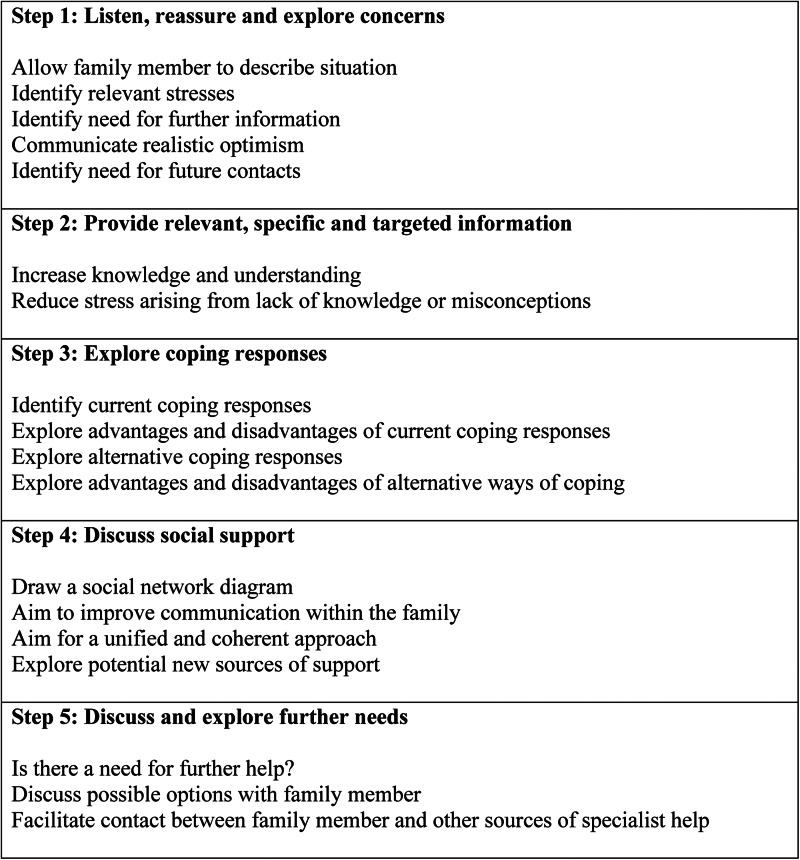
The 5-Step Method: 5 steps to support family members affected by addiction problems.

The intervention arm received SAFE, a contextually adapted version of the 5-Step Method (Nadkarni *et al*., 2019) (Fig. 1), delivered in five sessions over 10 weeks. The intervention was delivered face-to-face, at a mutually convenient venue (e.g. home). The control arm received EUC, which consisted of an information leaflet highlighting the nature and impact of alcohol-related problems, and sources of help for the AFM. In both arms, we referred participants with serious concerns (e.g. suicidal ideation) for further specialised treatment or urgent care.

### Interventionists

We recruited lay counsellors through referrals from community gatekeepers, advertisements and word-of-mouth publicity. The lay counsellors had no previous formal training in the field of mental health, were from the same community as the target population and were fluent in the vernacular languages. The lay counsellors were chosen after a thorough competency evaluation process and underwent rigorous training and supervision. Training entailed expert-led sessions on the 5-Step Method, followed by refresher trainings to address any identified learning needs. Supervision included both, individual and group, peer and expert-led supervision, where the counsellors discussed ratings on randomly selected recorded sessions on the SAFE therapy quality scale developed for the study, and also discussed feedback on challenges and barriers during sessions.

### Baseline assessments

All eligible participants who consented to participate were administered the following baseline assessments: socio-demographic questionnaire, Coping Questionnaire (CQ) (Orford *et al*., 2005) measuring the AFM's coping strategies, Symptom Rating Test (SRT) (Kellner and Sheffield, 1973) measuring physical and psychological symptoms, Family Member Impact (FMI) questionnaire (Orford *et al*., 2005) measuring the impact of the alcohol use on various aspects of the AFM's life, and Alcohol, Drugs and the Family Social Support Scale (ADF-SSS) (Toner and Velleman, 2014) measuring social support for the AFM. The CQ, SRT, FMI and ADF-SSS are standardised tools and have undergone rigorous psychometric testing for reliability and validity.

### Process indicators

Process evaluation was conducted to examine acceptability and feasibility of recruitment procedures and treatment, fidelity of implementation, moderation of impact and contextual factors associated with outcomes.

### Outcomes

The primary outcome was the difference in mean SRT scores at 3 months. The secondary outcomes were mean differences in CQ, FMI and ADF-SSS scores at 3 months. Lower scores on all the measures, except the ADF-SSS, denote fewer symptoms, fewer attempts at coping and fewer negative incidents impacting the family member, respectively (Kellner and Sheffield, 1973; Orford *et al*., 2005; Toner and Velleman, 2014). Other secondary outcomes included experience of physical and sexual violence. We conducted the outcome assessments between August and November 2016. The trial was completed in November 2016 when the 3-month outcome assessment ended.

### Sample size

We aimed to recruit 50 participants in each arm, which was assumed to be sufficient to examine feasibility and acceptability of the intervention and trial procedures.

### Statistical methods

Quantitative analyses were performed using STATA (version 14). The primary analysis was intention-to-treat at the 3-month endpoint, and secondary analyses at the 3-month endpoints, adjusted for baseline values. Per-protocol analysis was conducted in those who completed the trial. Missing outcome data were imputed by multiple imputation using baseline characteristics. An estimate of the effects of the intervention (relative to EUC), in terms of effect sizes are reported as crude and adjusted odds ratios (aOR) and standardized mean differences (s.m.d.) with 95% confidence intervals, as appropriate.

### Ethics

Ethical approval was sought from the Institutional Review Board of the host institution and the Indian Council of Medical Research (reference number: UB_2016_022).

## Results

**Fig. 2.**
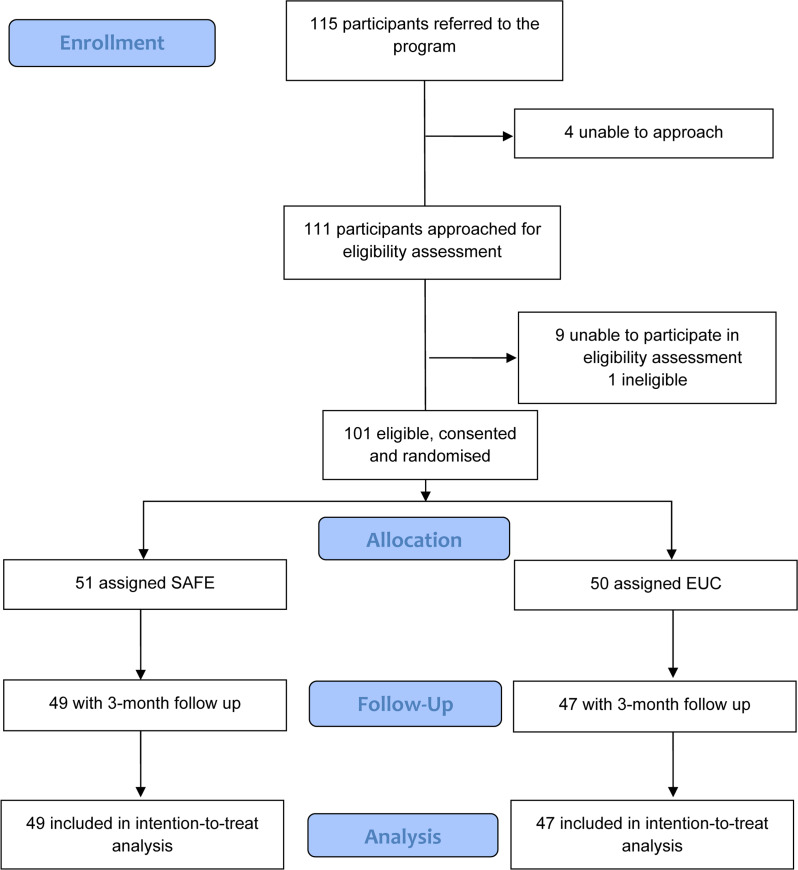
CONSORT flowchart for reporting pilot and feasibility trials'

Between May and August 2016, we recruited 101 participants in the study, of whom 51 were randomly allocated to the SAFE arm and 50 to the EUC arm (Fig. 2).

### Baseline characteristics

All of our participants were female apart from two who were both fathers of sons with drinking problems (Tables 1 and 2). Baseline characteristics were similar between the two arms, except for educational status and help-seeking by the drinking relative. A significantly higher proportion of those in the SAFE arm completed primary/secondary education compared to those in the EUC arm (90.2% *v.* 74%, *p* = 0.03). About 20% reported that their drinking relative had received treatment in the previous 3 months, and these were disproportionately recruited into the treatment arm (27% *v.* 10%, *p* = 0.02). Ninety-six participants (95%) completed outcome assessments (96% in the SAFE arm and 94% in the EUC arm).

**Table 1.** Baseline characteristics of trial participants by arm

	SAFE arm	EUC arm	*p* value
Age (years) [mean (s.d.)]	40.9 (11.4)	40.7 (9.9)	0.92
Gender			
Female	49 (96.1%)	50 (100%)	0.16
Male	2 (3.9%)	0	
Religion			
Hindu	49 (96.1%)	48 (96%)	0.98
Christian	2 (3.9%)	2 (4%)	
Marital status			
Married	48 (94.1%)	45 (90%)	0.44
Single or widowed	3 (5.9%)	5 (10%)	
Years of marriage [mean (s.d.)]	16.4 (10.7)	14.7 (7.8)	0.45
Educational status			0.03
No formal education	5 (9.8%)	13 (26%)	
Completed primary/secondary education	46 (90.2%)	37 (74%)	
Employment status			0.93
Employed (part time or full time)	22 (43%)	22 (44%)	
Not currently employed (unemployed, student, retired, home maker)	29 (56.8)	28 (56%)	
Relationship with the drinker			0.62
Parent	7 (13.7%)	12 (24%)	
Child	1 (2%)	1 (2%)	
Spouse	41 (80.4%)	35 (70%)	
Others (daughter-in-law, sister-in-law)	2 (3.9%)	2 (4%)	
Years of drinking (participant's relative) [mean (s.d.)]	15.2 (10.7)	14.3 (9.3)	0.65
Years of problematic drinking (participant's relative) (mean [s.d.)]	9.7 (9)	8.4 (6.7)	0.44
Satisfaction with relationship with the drinker (past 2 weeks)			
Satisfied	15 (31.2%)	14 (28.6%)	0.77
Neither satisfied nor dissatisfied	8 (16.7%)	11 (22.4%)	
Dissatisfied	25 (52.1%)	24 (49%)	
Experience of physical abuse	12 (23.5%)	16 (32%)	0.34
Experience of sexual abuse	7 (13.7%)	3 (6%)	0.29
Drinker knows of participant's involvement in the trial	7 (13.7%)	13 (26%)	0.12
Any form of help received by the participant	2 (3.9%)	4 (8%)	0.39
Any form of help received by the drinker	14 (27.4%)	5 (10%)	0.02

**Table 2.** Baseline characteristics of participants who completed outcome evaluation and those who were lost to follow-up

	Completed 3 months outcome assessment (*n* = 96)	Lost to follow-up (before 3 months outcome assessment) (*n* = 5)	*p* value
Age (years) [mean (s.d.)]	40.9 (10.8)	39.4 (7.7)	0.76
Gender			
Female	94 (97.9%)	5 (100%)	0.74
Male	2 (2.1%)	0	
Religion			
Hindu	92 (95.8%)	5 (100%)	0.64
Christian	4 (4.2%)	0	
Marital status			0.30
Married	89 (92.7%)	4 (80%)	
Single or widowed	7 (7.3%)	1 (20%)	
Years of marriage [mean (s.d.)]	15.6 (9.4)	16 (12.2)	0.94
Educational status			0.18
No formal education	16 (16.7%)	2 (40%)	
Completed primary/secondary education	80 (83.3%)	3 (60%)	
Employment status			0.09
Employed (part time or full time)	40 (41.7%)	4 (80%)	
Not currently employed (unemployed, student, retired, home maker)	56 (58.3%)	1 (20%)	
Relationship with the drinker			0.3
Parent	18 (18.8%)	1 (20%)	
Child	2 (2.1%)	0	
Spouse	73 (76%)	3 (60%)	
Others (daughter-in-law, sister-in-law)	3 (3.1%)	1 (20%)	
Years of drinking (participant's relative) [mean (s.d.)]	14.9 (9.8)	12.4 (14.0)	0.59
Years of problematic drinking (participant's relative) [mean (s.d.)]	9.2 (7.7)	7.4 (12.7)	0.62
Satisfaction with relationship with the drinker (past 2 weeks)			0.05
Satisfied	29 (31.5%)	0	
Neither satisfied nor dissatisfied	16 (17.4%)	3 (60%)	
Dissatisfied	47 (51.1%)	2 (40%)	
Experience of physical abuse	26 (27.1%)	2 (40%)	0.53
Experience of sexual abuse	9 (9.4%)	1 (20%)	0.67
Drinker knows of participant's involvement in the trial	19 (19.8%)	1 (20%)	0.99
Any form of help received by the participant	5 (5.2%)	1 (20%)	0.17
Any form of help received by the drinker	19 (19.8%)	0	0.27

### Experiences of family members

Across both arms, as reported by the AFMs at baseline, the average duration of the relatives' drinking was 14.7 (s.d. 10.0) years, and the average duration of problematic drinking behaviours was 9.1 (s.d. 7.9) years (Tables 1 and 2). At baseline, 28.7% of the participants expressed being ‘satisfied’ with their relationship, 18.8% reported being neither satisfied nor dissatisfied, and 48.5% expressed being dissatisfied. In total, 27.7% of participants reported being physically abused, and 9.9% reported being sexually abused. Only 5.9% of the participants had received any form of help in the past 3 months for the difficulties that they were experiencing in relation to the drinking behaviours of their relative. There were no significant differences between arms on satisfaction with relationship, experience of violence and help-seeking by the participant.

### Indicative impact of the intervention

At follow-up, the mean SRT, CQ, FMI and ADF-SSS scores were all higher in the SAFE arm compared to the EUC arm, however, the between-group adjusted mean differences were only statistically significant for ADF-SSS scores (AMD −6.05, 95% CI −10.98 to −1.12, *p* 0.02) (Tables 3–6). The SSCS model suggests that higher scores on each of the outcome measures (except ADF-SSS) suggest worse outcomes. Hence, the only outcome which went in the positive direction in the SAFE arm was ADF-SSS (i.e. social support). In comparing the change scores at 3 months (i.e. extent of change from baseline to outcome per arm), the change in CQ and ADF-SSS was greater in the SAFE arm, with the former changing in a negative direction (i.e. higher score indicating worse coping) and the latter changing in a positive direction (i.e. higher score indicating better social support). The change in SRT and FMI was greater in the EUC arm, with both changing in a positive direction (i.e. lower SRT score indicating lesser strain and lower FMI score indicating lesser stress). However, the between-group adjusted mean differences were not significant for any of these outcomes. In comparing sub-scale scores, there was a significant increase only in ‘engaged style’ of coping and ‘positive functional support’ in the SAFE arm. With regards to the experience of abuse, a greater (although non-significant) proportion of participants reported experiencing physical abuse in the EUC arm than in the SAFE arm (21.3% *v.* 12.5%, aOR 1.86, 95% CI 0.59–0.84, *p* = 0.29), while for sexual abuse, the difference was of 2.9% in the EUC arm *v.* 2.6% in the SAFE arm (aOR 1.27, 95% CI 0.08–21.28, *p* = 0.86).

**Table 3.** Intervention effect on SRT, CQ, FMI and ADFSSS scores at 3 months

	SAFE arm (*n* = 51) mean (s.d.)/proportion	EUC arm (*n* = 50) mean (s.d.)/proportion	Difference between means/odds ratio (95% CI)	*p* value	Intervention effect (adjusted mean difference/adjusted odds ratio^a^) (95% CI; *p* value)	*p* value
Primary outcome						
Strain (SRT score) [mean (s.d.)]	22.1 (14.5)	16.7 (15)	−5.41 (−11.66 to 0.84)	0.09	−4.01 (−10.82 to 2.80)	0.24
Secondary outcomes						
Coping (CQ score) [mean (s.d.)]	39.5 (13.5)	33.8 (17.9)	−5.76 (−12.54 to 1.02)	0.09	−3.55 (−10.00 to 2.91)	0.28
Stress (FMI score) [mean (s.d.)]	18.2 (10.8)	17.1 (13.3)	−1.122 (−6.14 to 3.89)	0.66	−0.49 (−4.47 to 5.45)	0.84
Social support (ADFSSS score) [mean (s.d.)]	24.5 (11.0)	17.1 (10.8)	−7.36 (−11.96 to −2.76)	0.002	−6.05 (−10.98 to −1.12)	0.02
Experience of physical abuse	6 (12.5%)	10 (21.3%)	1.52 (0.51–4.51)	0.45	1.86 (0.59–5.84)	0.29
Experience of sexual abuse	1 (2.6%)	1 (2.9%)	1.42 (0.08–24.03)	0.80	1.27 (0.08–21.28)	0.86

a Adjusted for educational status, any form of help received by the drinker at baseline and counsellor effects at baseline.

**Table 4.** Change scores (total score) at 3 months

	SAFE arm (*n* = 51) mean (s.d.)	EUC arm (*n* = 50) mean (s.d.)	Difference between means (95% CI)	p value	Intervention effect (adjusted mean difference^a^) (95% CI; *p* value)	*p* value
Primary outcome						
Change in SRT score	0.90 (10.24)	4.68 (10.47)	3.78 (−0.74 to 8.30)	0.10	3.57 (−1.05 to 8.19)	0.13
Secondary outcomes						
Change in CQ score	−3.95	−1.24	2.71 (−4.20 to 9.63)	0.44	2.17 (−4.88 to 9.21)	0.54
Change in FMI score	2.95	4.26	1.30 (−3.65 to 6.26)	0.60	1.73 (−3.29 to 6.76)	0.49
Change in ADFSSS score	−10.44	−6.16	4.28 (−1.13 to 9.69)	0.12	2.89 (−2.48 to 8.26)	0.29

a Adjusted for educational status, any form of help received by the drinker at baseline and counsellor effects at baseline.

**Table 5.** Intervention effect on SRT, CQ, FMI and ADFSSS sub-scale scores

Outcome	SAFE arm	EUC arm	*p* value
	Mean (95% CI)	Mean (95% CI)	
Symptom Rating Test			
Pre-treatment	23.25 (18.93–27.57)	21.48 (17.58–25.37)	0.54
Post treatment	22.15 (17.80–26.51)	16.74 (12.13–21.37)	0.09
Psychological			
Pre-treatment	14.37 (11.62–17.13)	13.04 (10.71–15.37)	0.46
Post treatment	13.91 (11.05–16.77)	9.58 (6.60–12.56)	0.037
Physical			
Pre-treatment	9.08 (7.38–10.78)	8.45 (6.77–10.13)	0.60
Post treatment	8.28 (6.73–9.84)	7.28 (5.57–8.99)	0.38
Coping Questionnaire			
Pre-treatment	35.26 (30.53–39.99)	31.36 (27.03–35.68)	0.22
Post treatment	39.52 (35.41–43.64)	33.76 (28.19–39.33)	0.09
Engaged coping			
Pre-treatment	19.02 (16.40–21.64)	16.55 (14.19–18.92)	0.16
Post treatment	22.21 (20.04–24.39)	17.45 (14.31–20.59)	0.012
Tolerant coping			
Pre-treatment	8.6 (6.84–10.36)	7.17 (5.41–8.93)	0.25
Post treatment	9.34 (7.60–11.09)	8.22 (6.12–10.31)	0.40
Withdrawal coping			
Pre-treatment	7.83 (6.70–8.96)	7.93 (6.82–9.05)	0.89
Post treatment	8.42 (7.20–9.64)	8.56 (7.20–9.93)	0.87
Family Member Impact questionnaire			
Pre-treatment	19.84 (16.71–22.97)	21.74 (17.94–25.53)	0.43
Post treatment	18.23 (15.05–21.41)	17.11 (13.11–21.10)	0.66
Worrying behaviour			
Pre-treatment	13.66 (11.61–15.70)	15.10 (12.75–17.44)	0.35
Post treatment	11.57 (9.60–13.54)	10.93 (8.34–13.53)	0.69
Active disturbance			
Pre-treatment	6.25 (5.02–7.47)	6.93 (5.46–8.40)	0.47
Post treatment	6.66 (5.17–8.15)	6.33 (4.75–7.92)	0.76
Alcohol Drugs and the Family Social Support Scale			
Pre-treatment	13.96 (10.08–17.83)	10.95 (7.57–14.34)	0.24
Post treatment	24.48 (21.21–27.75)	17.12 (13.79–20.44)	0.002
Positive functional support			
Pre-treatment	15.29 (12.35–18.23)	12.55 (9.60–15.50)	0.19
Post treatment	22.39 (19.87–24.91)	16.41 (13.54–19.27)	0.002
Positive alcohol, drugs and families specific support			
Pre-treatment	4.69 (3.46–5.92)	4.51 (3.17–5.85)	0.84
Post treatment	7.85 (6.67–9.03)	6.4 (5.17–7.63)	0.09
Negative alcohol, drugs and families specific support			
Pre-treatment	5.55 (3.94–7.16)	5.69 (3.86–7.53)	0.90
Post treatment	5.75 (4.13–7.36)	5.44 (3.62–7.26)	0.80

**Table 6.** Intervention effect on change in SRT, CQ, FMI and ADFSSS sub-scale scores

Outcome	SAFE arm	EUC arm	*p* value
	Mean (95% CI)	Mean (95% CI)	
Symptom Rating Test			
Psychological	0.43 (−1.72 to 2.58)	2.98 (0.53–5.42)	0.12
Physical	1.04 (−0.29 to 2.37)	1.35 (0.29–2.42)	0.71
Coping Questionnaire			
Engaged coping	−2.81 (−5.60 to 0.02)	−0.71 (−4.13 to 2.71)	0.33
Tolerant coping	−0.66 (−2.32 to 1.00)	−0.42 (−2.16 to 1.32)	0.84
Withdrawal coping	−0.45 (−1.68 to 0.78)	−0.16 (−1.53 to 1.2)	0.75
Family Member Impact questionnaire			
Worrying behaviour	2.65 (0.37–4.93)	4.28 (1.68–6.89)	0.34
Active disturbance	0.16 (−1.18 to 1.50)	−0.13 (−1.55 to 1.29)	0.76
Alcohol Drugs and the Family Social Support Scale			
Positive functional support	−6.72 (−9.69 to 3.75)	−3.75 (−6.74 to 0.76)	0.16
Positive alcohol, drugs and families specific support	−2.93 (−4.23 to 1.63)	−1.91 (−3.21 to 0.61)	0.26
Negative alcohol, drugs and families specific support	−0.022 (−1.70 to 1.65)	0.38 (−1.34 to 2.10)	0.74

### Acceptability and feasibility of the intervention

One hundred fifteen participants were referred to the trial; 95 were referred by community gatekeepers and 20 (17.4%) were self-referred (Tables 7 and 8). One hundred eleven were approached to assess eligibility, and 102 completed eligibility assessment. One hundred one participants were eligible, consented to randomisation and completed baseline assessments. Of the 51 participants who entered treatment, 40 (78%) completed treatment/had a planned discharge (i.e. all five sessions). There were no statistically significant differences in baseline characteristics between those who completed treatment and those who did not. Of the remainder (*n* = 11), three dropped out before the first session, five dropped out after the second session and three dropped out after the third session. The average number of sessions conducted was 4.25. The mean duration of sessions was 53 min. The mean duration reduced from session 1 to session 3 before increasing again in the final session. For the majority of the participants, the first session was conducted in the clinic, and this proportion gradually reduced over the duration of treatment, with most sessions being conducted at participants' homes by the fourth session. Finally, the mean number of days between sessions increased as the treatment progressed from session 1 to session 4 (from 16 to 31 days). There were seven serious adverse events (three in the SAFE arm, and four in the EUC arm) reported over the trial period. All of the serious adverse events reported were violence perpetrated by the drinking relative (and in two cases, violence was reported to be experienced by other family members).

**Table 7.** Details of counselling sessions

Session	Number of participants *n* (%)	Mean duration in minutes (s.d.)	Location *n* (%)		Mean number of days between sessions
Session 1	48 (94.1%)	57.1 (14.0)	Home	23	
			Clinic	4	
			Other community setting	21	
			Phone	0	
Session 2	46 (90.2%)	55.4 (11.5)	Home	21	16.22 days
			Clinic	6	
			Other community setting	19	
			Phone	0	
Session 3	43 (84.3%)	49.5 (9.4)	Home	19	15.88 days
			Clinic	3	
			Other community setting	21	
			Phone	0	
Session 4	40 (78.4%)	56.2 (10.1)	Home	18	19.15 days
			Clinic	4	
			Other community setting	18	
			Phone	0	
Session 5	40 (78.4%)	47.4 (11.3)	Home	20	12.93 days
			Clinic	4	
			Other community setting	16	
			Phone	0	
Booster session	36 (70.6%)	6.3 (2.7)	Home	9	
			Clinic	1	
			Other community setting	3	
			Phone	23

**Table 8.** Details of counselling sessions in treatment completers and dropouts

	All participants	Dropped out	Completed treatment or planned discharge
Mean number of sessions	4.25 sessions	1.54 sessions	5 sessions
Mean duration (minutes) of treatment	53.31 min	51.06 min	53.51 min

## Discussion

Our pilot RCT has demonstrated that the intervention has good acceptability and feasibility, but has only shown positive indicative impact on one of the social support measures: positive functional support. These findings are consistent with our previous work, where we conducted a case series with a uncontrolled treatment cohort of AFMs, and again found positive changes only in social support [in fact, that study showed positive changes in both positive functional support and positive alcohol, drugs and families specific support, as well as an increase in strain and engaged coping (Nadkarni *et al*., 2019)]. However, these findings are inconsistent with most other 5-Step Method work outside India. In almost all major evaluations of the intervention, the 5-Step Method has led to significant reductions in strain, and a reduction in engaged and tolerant coping (Copello *et al*., 2010a, 2010b). A key question therefore is how to account for these differences. One potential attribution is that they might be related to how culture influences the experiences of AFMs. It has been suggested that different cultures and contexts influence the accumulated burden that AFMs hold, and the social support available for them; and that these differences may influence the core AFM experience (Orford, 2017). This is especially true for our setting where our previous work has demonstrated both high levels of burden and the inaccessibility of effective social support (Church *et al*., 2018).

The high uptake and completion of the intervention suggest that it was acceptable to family members; and indicate that there was a felt need for the intervention, it was possible to identify AFMs through gatekeeper or self-referrals, and it was acceptable to participants. Also, it implies that a program focused on AFMs can be implemented in this context with good initiation and follow-up rates. Further, in resource-constrained settings, it is feasible and acceptable to train lay counsellors to deliver a psychosocial intervention for families in community settings. From the perspective of evaluation research, it is possible to efficiently implement procedures that are critical in conducting a definitive RCT of the SAFE intervention, which had been our planned next step.

However, despite the fact that feasibility studies are not powered to assess effectiveness, our quantitative findings raise several questions. Does the fact that, for the participants receiving SAFE, stress and strain did not significantly reduce, and there was an increased use of coping methods which studies in other countries have suggested may be less helpful ways of coping, suggests that the intervention may (or does) not work in our context? Although our study cannot empirically answer that question, we can speculate about a number of potential explanations. First, the philosophical focus of the intervention may not be the best ‘cultural fit’ for the Indian context. The intervention is focused solely on empowering family members in their own right, and is thus a significant shift from the traditional neglect or token involvement of family members to direct engagement/focus on family members. In a ‘collectivist’ culture such as India which ascribes greater importance to the family as a unit, the focus of the SAFE intervention on the individual may have led to a mismatch. To illustrate this point, we would like to draw upon one of our anecdotal experiences from our process and qualitative work. AFMs strongly asserted that although they were burdened, their primary interest was not to receive help for themselves; instead their preference was that their drinking relative should engage in any form of treatment, and change his drinking behaviours, which would make ‘all their problems go away’. Hence, it may be that AFMs in our context retained a focus on the drinking relative and did not see the appropriateness or the possible usefulness of an intervention which might exclusively support the AFMs. The intervention was developed and has been used elsewhere in order to help AFMs in their own right by focusing on their coping and support systems; and it is possible that the AFMs whom we recruited in this context did not want this or understand its potential helpfulness, and instead wanted an intervention which would persuade their drinking relatives to seek treatment. As a result, by focusing on how they as AFMs were experiencing the impact of their drinking relative, our intervention may have increased stress and strain in family members.

Another possibility could be that the health of these AFMs did not improve because their situation remained the same: the drinker had not stopped drinking (which was their primary perceived need), and hence their perception might have been that ‘nothing had changed’ and hence they did not change either.

A related point is that this sample scored considerably lower on especially stress (FMI), but also on strain (SRT) than have other samples drawn from other countries and cultures [pre-treatment FMI scores in other countries are typically in the range of 30, whereas pre-treatment FMI scores in this sample were 19.84 (SAFE arm) and 21.74 (EUC arm); pre-treatment SRT scores range from 28.5 to 36.8, whereas here they were 23.25 (SAFE) and 21.48 (EUC)] (Copello *et al*., 2010a, 2010b). The culture in India creates a situation where women are made to ‘accept their lot’ and as a result accept that they have to live with men who drink too much, and that sometimes they suffer as a result of that; and hence many women are relatively fatalistic about this (Stanley, 2012). Perhaps, by getting AFMs to undergo five sessions of a counselling intervention which got them to reflect on this situation, and by discussing change, it may have allowed these AFMs to realise that the impact and the effects on themselves were even worse than they had previously considered. Related to this, the intervention focuses on ‘alternatives to current ways of coping’ but in the study context it is possible that many of these AFMs might have felt that their alternatives were so limited that they could not change their situations. Hence our intervention may have raised awareness and self-reflection (e.g. regarding the negative impacts and their own responses), without alleviating any problems (e.g. getting the husband to stop drinking; or generating reasonable alternatives to current ways of coping). Again, there is some corroboration for this view, in that the style of coping which increased in this sample was engaged coping, one in which the focus is very much on the drinker and the drinking.

One further alternative explanation could be that the brief nature of our intervention was not adequate to lead to any longer-term positive changes in AFMs, especially in a context where ‘talking therapies’ are a novel and quite alien concept (Murthy, 2014).

A different explanation might relate to the competency of counsellors in delivering the 5-Step Method, which in previous studies has been delivered by specialists (Copello *et al*., 2009; Velleman *et al*., 2011). It could be argued that the treatment quality could have been affected because the delivery agents were not trained professionals, but were lay counsellors. However, there is an extensive evidence base of the effectiveness of non-specialist health worker-delivered mental health interventions in LMICs (van Ginneken *et al*., 2013; Nadkarni *et al*., 2017; Patel *et al*., 2017; Singla *et al*., 2017).

Our recruitment strategy may also explain the characteristics of participants, and the mixed results we found. We recruited participants through self and gatekeeper referrals, which meant that our sample would have included both those who were treatment seekers, and those who were not seeking treatment for their experiences or problems. Recruiting non-treatment seekers meant that there may have been a sub-group of participants who were less likely to fully engage in treatment, and report positive change (the lower levels of reported stress and strain offer some corroboration for this). Finally, it may be that the quantitative measures we have used are not culturally sensitive and adequate enough to capture actual and relevant changes that AFMs experience in their lives.

The second fundamental question is about the differential direction of change across the various domains of the experience of AFMs: Why did only social support scores increase? One of the predictors of future help-seeking is past experience of accessing help. In an Australian study, AFMs reported that positive help-seeking experiences in the past made them more likely to access services (McCann and Lubman, 2018). Our intervention provided AFMs with a safe space where they felt heard, and attempted to empower them to cope better; but for many this was the first such help that they had ever accessed. It is possible that the positive experiences that AFMs experienced during the SAFE sessions may have increased the likelihood of further help-seeking and hence led to an increase in social support scores.

Despite the improvement in perceived support, there was no positive change in the other domains of the SSCS model, and this could suggest a number of possibilities. One is that positive impact in this sample may only be seen over a longer time span. Alternatively, the available social support structures were perhaps not adequate, in terms of whether these were accessible, or even the quality of support offered. Hence, although there may have been increased support, the impact of the support in terms of improving the situation of AFMs may not have been positive. Given the relatively inextricable contexts that AFMs come from, this increased support may have not led to any significant change in the stress, strain and coping of AFMs. Finally, the increased support reported by the AFMs could actually be the support provided by lay counsellors through the SAFE intervention. This explanation is also likely given that our previous work has demonstrated the inaccessibility of social support (Church *et al*., 2018).

To conclude, there are five major implications of our results. First, the fast recruitment and high acceptance rates for the intervention demonstrate that there is a great need for interventions aimed at AFMs in this context; and the high adherence rates demonstrate that the AFMs found the experience of receiving the intervention relevant and helpful. Both our anecdotal and qualitative data suggest that the large majority of the AFMs who received the intervention felt that they needed an intervention, stated that they felt better as a result of it and appreciated their meetings with their counsellors. These findings then reinforce what is known from other studies in India and elsewhere: that AFMs carry a high burden and a there is a major need for interventions to reduce this burden.

Second, we now have a better understanding of how this intervention worked in the setting. We found that AFMs in both arms showed at the time of the 3-month follow-up greater levels of stress and strain than at baseline, and that those in the intervention arm both increased the amount of engaged coping they used and reported greater changes in positive social support. Clearly this study was not powered to demonstrate significant results, but these preliminary findings strongly suggest that this intervention did not lead to expected changes – that is to reductions in especially strain (symptoms) and in AFMs generally feeling more empowered and more ‘in control’ of their situation. The rise in engaged coping suggests instead that, by focusing their attention on their situations, the intervention caused these AFMs to attempt to intervene at an even higher level than they had done before, yet with them still not being able to change the situation.

This relates to a third implication; that the intervention did not address the most pressing problem for these AFMs, which was to ensure that their relative with a drinking problem received effective treatment and stopped drinking. In some ways this is not an uncommon issue within the 5-Step Method, where many AFMs come for help with a focus on their problem relative. But in this context, it proved extremely challenging to assist these AFMs to see that if they were able to change their own behaviour, and to develop alternative ways of dealing with the problem, this might improve relationships in the family, and reduce their stress and strain. Our counsellors reported that many AFMs seemed extremely ‘stuck’, or alternatively lived in contexts where change seemed exceedingly difficult, and found it very problematic to generate alternative coping strategies, even with considerable help from the counsellors. One solution to this might be to broaden the scope of such interventions, for instance, planning horizontal approaches to care, primarily addressing the needs of both, the relative with the drinking problem and their AFMs. Keeping in line with this proposed shift of focus, there is scope to explore the relevance of offering 5-Step Method with a supplemented focus on the relative with the drinking problem, as well as other systemic and collaborative approaches to family-based interventions such as the Community Reinforcement and Family Training where family members and their relative with the drinking problem are jointly engaged (Copello *et al*., 2005).

Fourth, the findings from this pilot RCT corroborated our earlier findings from both our formative work and other work undertaken within India, which is that domestic violence is a major and unresolved problem for many AFMs. Some of this violence is alcohol-related; but some is simply endemic to a culture where violence against women is an accepted and often largely ignored part of the landscape. Further work is needed to create interventions for AFMs which address this wide range of needs and complex situations (Copello *et al*., 2006). This point leads into a wider implication from this study: how socio-cultural factors influence the impact of interventions. Almost all of our participants were women, who reported experiences which clearly emphasised how patriarchal structures continue to disadvantage them (e.g. the high prevalence of violence). Although the focus of this intervention was on empowerment, it was not uncommon for women to express their needs through the lens of their partners and children, to report multiple burdens as a result of assuming the primary caregiver's role, to report various expressions of violence, and to report poor social support (Church *et al*., 2018; Nadkarni *et al*., 2019). It is important to note that these experiences are not unique to Goa: previous studies with AFMs have found similar stories, particularly from women (Rey *et al*., 2010; Stanley, 2012). This interplay of gender with the experience of being an AFM suggests the need for the inclusion of other perspectives (e.g. a feminist understanding) in planning psychosocial interventions (Orford *et al*., 2010b). An example of this feminist approach is from a Mexican qualitative study with AFMs, where female participants experienced positive change in their lives by learning how to change their locus of control (moving from ‘women being objects to subjects of their own destiny’) (Rey *et al*., 2010).

Some limitations of our study include one of the ways in which we recruited participants (through self-referrals), which may result in a non-representative sample, as participants who volunteer for a trial may disproportionately possess characteristics related to the outcome. Another important limitation was the reliance on self-reports for outcome evaluation, which introduce an element of subjectivity, and are prone to response bias.

The following characteristics are key strengths of our study. First, we focused on a target population that is considered to be a ‘hidden’ or ‘silent’ and hard-to-reach group. The primary platform through which we recruited our sample was the community, which expanded the potential reach of our study, and improved the representativeness of our sample. Second, to the best of our knowledge, this is the first study that has systematically examined an evidence-based intervention for AFMs ‘in their own right’ in the Indian context. Third, our community-based participatory research approach allowed for a range of community stakeholders to inform the design, conduct and evaluation of the intervention.

## Conclusion

Our study aids the process of translational research by adopting frameworks that have an established evidence base and implementing these frameworks in a culturally appropriate manner to newer underserved populations. Given our mixed findings, further work is required to unpack how the intervention affects AFMs and what further adaptations are required to make the intervention relevant to the Indian context.

## Supplementary material

For supplementary material accompanying this paper visit http://dx.doi.org/10.1017/gmh.2022.41. click here to view supplementary material

## References

[ref1] Benegal V, Chand PK and Obot IS (2009) Packages of care for alcohol use disorders in low-and middle-income countries. PLoS Medicine 6, e1000170. 19859536 10.1371/journal.pmed.1000170 PMC2761617

[ref2] Church S, Bhatia U, Velleman R, Velleman G, Orford J, Rane A and Nadkarni A (2018) Coping strategies and support structures of addiction affected families: a qualitative study from Goa, India. Families, Systems, & Health 36, 216. 10.1037/fsh0000339 29902038

[ref3] Copello A and Orford J (2002) Addiction and the family: is it time for services to take notice of the evidence? Addiction 97, 1361–1363. 12410776 10.1046/j.1360-0443.2002.00259.x

[ref4] Copello AG, Velleman RD and Templeton LJ (2005) Family interventions in the treatment of alcohol and drug problems. Drug and Alcohol Review 24, 369–385. 16234133 10.1080/09595230500302356

[ref5] Copello AG, Templeton L and Velleman R (2006) Family interventions for drug and alcohol misuse: is there a best practice? Current Opinion in Psychiatry 19, 271–276. 16612212 10.1097/01.yco.0000218597.31184.41

[ref6] Copello A, Templeton L, Orford J, Velleman R, Patel A, Moore L, MacLeod J and Godfrey C (2009) The relative efficacy of two levels of a primary care intervention for family members affected by the addiction problem of a close relative: a randomized trial. Addiction 104, 49–58. 19133888 10.1111/j.1360-0443.2008.02417.x

[ref7] Copello A, Templeton L, Orford J and Velleman R (2010a) The 5-Step Method: evidence of gains for affected family members. Drugs: Education, Prevention and Policy 17, 100–112.

[ref8] Copello A, Templeton L and Powell J (2010b) The impact of addiction on the family: estimates of prevalence and costs. Drugs: Education, Prevention and Policy 17, 63–74.

[ref9] Craig P, Dieppe P, Macintyre S, Michie S, Nazareth I and Petticrew M (2008) Developing and evaluating complex interventions: the new Medical Research Council guidance. BMJ 337, a1655. 18824488 10.1136/bmj.a1655 PMC2769032

[ref10] D'costa G, Nazareth I, Naik D, Vaidya R, Levy G, Patel V and King M (2006) Harmful alcohol use in Goa, India, and its associations with violence: a study in primary care. Alcohol and Alcoholism 42, 131–137. 17172257 10.1093/alcalc/agl103

[ref11] Gururaj G, Murthy P, Girish N and Benegal V (2011) Alcohol Related Harm: Implications for Public Health and Policy in India. Bangalore: NIMHANS.

[ref12] Kellner R and Sheffield BF (1973) A self-rating scale of distress. Psychological Medicine 3, 88–100. 4692494 10.1017/s0033291700046377

[ref13] Manthey J, Shield KD, Rylett M, Hasan OS, Probst C and Rehm J (2019) Global alcohol exposure between 1990 and 2017 and forecasts until 2030: a modelling study. Lancet 393, 2493–2502. 31076174 10.1016/S0140-6736(18)32744-2

[ref14] Mattoo SK, Nebhinani N, Kumar BA, Basu D and Kulhara P (2013) Family burden with substance dependence: a study from India. The Indian Journal of Medical Research 137, 704. 23703337 PMC3724250

[ref15] McCann TV and Lubman DI (2018) Help-seeking barriers and facilitators for affected family members of a relative with alcohol and other drug misuse: a qualitative study. Journal of Substance Abuse Treatment 93, 7–14. 30126543 10.1016/j.jsat.2018.07.005

[ref16] Murthy RS (2014) Mental health initiatives in India (1947–2010)*. Social Work in Mental Health: Contexts and Theories for Practice 5, 28.

[ref17] Nadkarni A, Weiss HA, Weobong B, McDaid D, Singla DR, Park AL, Bhat B, Katti B, McCambridge J, Murthy P, King M, Wilson GT, Kirkwood B, Fairburn CG, Velleman R and Patel V (2017) Sustained effectiveness and cost-effectiveness of counselling for alcohol problems, a brief psychological treatment for harmful drinking in men, delivered by lay counsellors in primary care: 12-month follow-up of a randomised controlled trial. PLoS Medicine 14, e1002386. 28898239 10.1371/journal.pmed.1002386 PMC5595289

[ref18] Nadkarni A, Bhatia U, Velleman R, Orford J, Velleman G, Church S, Sawal S and Pednekar S (2019) Supporting addictions affected families effectively (SAFE): a mixed methods exploratory study of the 5-step method delivered in Goa, India, by lay counsellors. Drugs: Education, Prevention and Policy 26, 195–204.

[ref19] Orford J (2017) How does the common core to the harm experienced by affected family members vary by relationship, social and cultural factors? Drugs: Education, Prevention and Policy 24, 9–16.

[ref20] Orford J, Templeton L, Velleman R and Copello A (2005) Family members of relatives with alcohol, drug and gambling problems: a set of standardized questionnaires for assessing stress, coping and strain. Addiction 100, 1611–1624. 16277623 10.1111/j.1360-0443.2005.01178.x

[ref21] Orford J, Copello A, Velleman R and Templeton L (2010a) Family members affected by a close relative's addiction: the stress-strain-coping-support model. Drugs: Education, Prevention and Policy 17, 36–43.

[ref22] Orford J, Velleman R, Copello A, Templeton L and Ibanga A (2010b) The experiences of affected family members: a summary of two decades of qualitative research. Drugs: Education, Prevention and Policy 17, 44–62.

[ref23] Orford J, Natera G, Copello A, Atkinson C, Mora J, Velleman R, Crundall I, Tiburcio M, Templeton L and Walley G (2013a) Coping with Alcohol and Drug Problems: The Experiences of Family Members in Three Contrasting Cultures. UK: Routledge.

[ref24] Orford J, Velleman R, Natera G, Templeton L and Copello A (2013b) Addiction in the family is a major but neglected contributor to the global burden of adult ill-health. Social Science & Medicine 78, 70–77. 23268776 10.1016/j.socscimed.2012.11.036

[ref25] Patel V (2007) Alcohol use and mental health in developing countries. Annals of Epidemiology 17, S87–S92.

[ref26] Patel V, Kirkwood BR, Pednekar S, Pereira B, Barros P, Fernandes J, Datta J, Pai R, Weiss H and Mabey D (2006) Gender disadvantage and reproductive health risk factors for common mental disorders in women: a community survey in India. Archives of General Psychiatry 63, 404–413. 16585469 10.1001/archpsyc.63.4.404

[ref27] Patel V, Weobong B, Weiss HA, Anand A, Bhat B, Katti B, Dimidjian S, Araya R, Hollon SD and King M (2017) The Healthy Activity Program (HAP), a lay counsellor-delivered brief psychological treatment for severe depression, in primary care in India: a randomised controlled trial. The Lancet 389, 176–185. 10.1016/S0140-6736(16)31589-6 PMC5236064 27988143

[ref28] Pillai A, Nayak MB, Greenfield TK, Bond JC, Nadkarni A and Patel V (2013) Patterns of alcohol use, their correlates, and impact in male drinkers: a population-based survey from Goa, India. Social Psychiatry and Psychiatric Epidemiology 48, 275–282. 22752108 10.1007/s00127-012-0538-1 PMC3529206

[ref29] Rane A, Church S, Bhatia U, Orford J, Velleman R and Nadkarni A (2017) Psychosocial interventions for addiction-affected families in low and middle income countries: a systematic review. Addictive Behaviors 74, 1–8. 28554034 10.1016/j.addbeh.2017.05.015

[ref30] Ray GT, Mertens JR and Weisner C (2009) Family members of people with alcohol or drug dependence: health problems and medical cost compared to family members of people with diabetes and asthma. Addiction 104, 203–214. 19149814 10.1111/j.1360-0443.2008.02447.x PMC2896239

[ref31] Rey GN, Mora-Ríos J, Sainz MT and Aguilar PM (2010) An international perspective: constructing intervention strategies for families in Mexico. Drugs: Education. Prevention and Policy 17, 193–202.

[ref32] Silva MC, Gaunekar G, Patel V, Kukalekar DS and Fernandes J (2003) The prevalence and correlates of hazardous drinking in industrial workers: a study from Goa, India. Alcohol and Alcoholism 38, 79–83. 12554613 10.1093/alcalc/agg016

[ref33] Singla DR, Kohrt BA, Murray LK, Anand A, Chorpita BF and Patel V (2017) Psychological treatments for the world: lessons from low-and middle-income countries. Annual Review of Clinical Psychology 13, 149–181. 10.1146/annurev-clinpsy-032816-045217 PMC5506549 28482687

[ref34] Stanley S (2012) Interactional dynamics in alcohol-complicated marital relationships: a study from India. Marriage & Family Review 48, 583–600.

[ref35] Toner P and Velleman R (2014) Initial reliability and validity of a new measure of perceived social support for family members of problem substance users. Addiction Research & Theory 22, 147–157.

[ref36] van Ginneken N, Tharyan P, Lewin S, Rao GN, Meera S, Pian J, Chandrashekar S and Patel V (2013) Non-specialist health worker interventions for the care of mental, neurological and substance-abuse disorders in low-and middle-income countries. Cochrane Database of Systematic Reviews, Issue 11. Art. No.: CD009149. 10.1002/14651858.CD009149.pub2 24249541

[ref37] Velleman R, Arcidiacono C, Procentese F, Copello A and Sarnacchiaro P (2008) A 5-step intervention to help family members in Italy who live with substance misusers. Journal of Mental Health 17, 643–655.

[ref38] Velleman R, Orford J, Templeton L, Copello A, Patel A, Moore L, Macleod J and Godfrey C (2011) 12-Month follow-up after brief interventions in primary care for family members affected by the substance misuse problem of a close relative. Addiction Research & Theory 19, 362–374.

